# Editorial: Pediatric TBI - Current State of the Art and Future Perspective

**DOI:** 10.3389/fneur.2021.649676

**Published:** 2021-02-18

**Authors:** Elham Rostami, Anthony Figaji, P. David Adelson

**Affiliations:** ^1^Department of Neuroscience Section of Neurosurgery, Uppsala University, Uppsala, Sweden; ^2^Department of Neurosurgery, Karolinska Institutet, Stockholm, Sweden; ^3^Division of Neurosurgery and Neurosciences Institute, University of Cape Town, Cape Town, South Africa; ^4^Department of Neurosurgery, Phoenix Children's Hospital, Phoenix, AZ, United States

**Keywords:** traumatic brain injury, pediatric brain injury, neuromonitoring, ICP, CBF

Traumatic Brain Injury (TBI) remains a leading cause of death and disability in children worldwide and those who survive may suffer long-term cognitive and physical disabilities, The mechanism of injury often differs between high income and low- and middle-income countries, where road traffic accidents and interpersonal violence are comparatively more common. In the publication of Riemann et al. using data collected through CENTER-TBI which is a multicenter study conducted in Europe and Israel, they found that road-traffic incidents were the most common cause of injury overall and those admitted to ICU, while incidental falls were most common in patients admitted to the hospital wards. The overall mortality rate was 3% and the rate of unfavorable outcome 10%, where Glasgow Coma Score (GCS) and the occurrence of secondary insults were identified as independent predictors for an unfavorable outcome. Monitoring the injured brain is crucial in the management of TBI in order to prevent and detect secondary insults. Brain imaging and multimodal neuromonitoring in adults with TBI have improved diagnostics and management of these patients but there is limited experience in the pediatric population. As Appavu et al. describe in their paper methods of monitoring real-time cerebral physiology are also needed in the pediatric population to better understand when secondary brain injury develops and what treatment strategies may alleviate or prevent such injury. They discuss several different emerging technologies to better understand intracranial pressure (ICP), cerebral blood flow, metabolism, oxygenation, and electrical activity. While recent guidelines recommend ICP monitoring and a treatment threshold of 20 mmHg for 5 min, Hornshoj Pedersen et al. highlight the lack of data on normal ICP in healthy children to understand and guide treatment of TBI. Although age-differentiated ICP thresholds in pediatric TBI are needed, only one study reported this and it did not correlate with outcome. The issue with age-differentiated thresholds is also discussed by Rostami et al. regarding CBF. Monitoring of CBF and autoregulation following TBI is crucial. However, there is still lack of fundamental knowledge about normal physiology in children for both across the age spectrum and between genders. Few studies available report on differences across the age range. Following TBI, low initial CBF correlates with poor outcome, as does impaired cerebral pressure autoregulation, but the relationship between the two still needs clarification. Current studies are few and mainly based on small number of patients between the broad age span of 0–18 years. Larger studies across narrower age ranges are needed. The age range affects physiology, mechanism of injury, and outcome. For example, TBI outcome is age dependant: children under the age of 4–5 years have the worse outcome and the rates of TBI-related emergency department visits in this group have increased by more than 50% in US during recent years. Children under the age of 4 years are also the group mostly at risk of abusive head trauma (AHT). O'Meara et al. highlight the difficulty in early identification of AHT and how its incidence is underestimated. Pediatric TBI studies often exclude AHT, which limits the generalization of research to this population. Emerging imaging modalities and biomarkers may improve the diagnostic workup, but these must also be applied to the acute phase of AHT. Use of TBI models may improve our understanding of the underlying pathology. Martin et al. showed in a transgenic mouse model that genes important in age-related neurodegenerative diseases in humans have significant impact on mortality and morbidity after early-life brain injury and may influence adult-onset neurodegenerative disease during aging.

Pediatric TBI also has long-lasting social, cognitive, physiological, and neurological impairments that can be hard to predict. Lindsey et al. highlight the importance of MRI in the acute management of pediatric TBI but most importantly its particular relevance for the sequential assessment of long-term consequences from injuries sustained to the developing brain. Different MRI modalities can reveal different aspects of the injury such as morphological changes in gray matter volume and cortical thickness, microstructural integrity of white matter, metabolic and neurochemical alterations in the brain, functional changes that occur as a result of structural damage, and typical developmental processes. There are few studies published in this field, and these demonstrate considerable heterogeneity in post-injury outcome. Larger sample sizes and multi-center future studies may identify key predictors of outcome following TBI.

One common neurological complication of severe TBI in children is spasticity, which may develop in up to 38% of patients within the first 12 months post injury. Enslin et al. discuss the importance of early identification, because late intervention has limited effect and major corrective surgery may then be needed. Most of the current data originates from stroke research. The review covers underlying pathophysiology as well as available prevention and management of spasticity.

Endocrinopathies (endocrine system disorders) may be caused by injury to the hypothalamic-pituitary axis/region. This is often overlooked and rarely reported but if left untreated, it may lead to subsequent health issues, hence the importance of early detection in pediatric TBI survivors.

Ortiz et al. found that children with a TBI diagnosis had 3.22 times greater risk of a central endocrine diagnosis compared with the general population (±0.28). This was also more common in females compared to males (64.1 vs. 35.9%). They suggest physicians managing pediatric TBI follow-up care should include preventive screening for endocrine disorders.

In this Research Topic we highlight the current knowledge in several key areas of pediatric TBI as well as gaps that can be the focus of future research.

## Author Contributions

All authors listed have made a substantial, direct and intellectual contribution to the work, and approved it for publication.

## Conflict of Interest

The authors declare that the research was conducted in the absence of any commercial or financial relationships that could be construed as a potential conflict of interest.

